# Happiness, Psychology, and Degrees of Realism

**DOI:** 10.3389/fpsyg.2016.01148

**Published:** 2016-08-03

**Authors:** Andrea Lavazza

**Affiliations:** Neuroethics, Centro Universitario InternazionaleArezzo, Italy

**Keywords:** happiness, well-being, realism, positive psychology, happiness satisfaction, positive psychology intervention, hedonic well-being

## Abstract

The recent emphasis on a realist ontology that cannot be overshadowed by subjectivist or relativist perspectives seems to have a number of consequences for psychology as well. My attempt here is to analyse the relationship between happiness as a state of the individual and the states of the external world and the brain events related to (or, in some hypotheses, causally responsible for) its occurrence. It can be maintained that different degrees of realism are suitable to describe the states of happiness and this fact might have relevant psychological implications, namely for the so-called positive psychology. This is especially true now that there are methods available to induce subjective states of happiness unrelated to the external conditions usually taken to be linked to such states.

## Introduction

The recent emphasis on the inescapable presence of a realist ontology that cannot be overshadowed by subjectivist or relativist perspectives (e.g., Harman, 2010; Gabriel, 2011; Ferraris, 2014, 2015) has sparked a debate that has only marginally touched on the field of experimental science. This is due to the easily understandable reason that the conditions of empirical research, while being mostly implicit, are entirely realist in the proper and most intuitive sense. If there were no external reality independent of the knowing mind—a reality that can be investigated insofar as it is accessible by our senses and our tools, predictable in its change and mostly interpretable according to law-like regularities—scientific inquiry would be neither practicable nor would it give us knowledge. And in any case this knowledge would not be effective and practical in the sense of allowing for correct predictions about future states of the world. Laboratory results seem to constitute the best refutation of the idea—expressed by Friedrich Nietzsche and attributed to anti-realists in general—that there are no facts, only interpretations (as Nietzsche wrote in Fragment 481 of *The Will to Power*).

Yet, the epistemological debate on scientific realism is very complex and is strongly interwoven with the vicissitudes of the concept of naturalism (De Caro and Macarthur, 2004, 2010). “New realism” principally states that what exists (ontology) is prioritary and more important than what we know about what exists (epistemology), and that the real is “unamendable” because it does not depend on our conceptual schemes or our linguistic practices. And this view easily, but not always, tends to conflate with scientific naturalism. This is in contrast to the general postmodern conception that all reality is socially constructed and infinitely manipulable; truth—in the culturalist perspective—is a useless notion, because solidarity is more important than objectivity (Ferraris, 2014).

This is the background of the topic that I want to develop here, which is also interesting for its implications in terms of empirical psychology and practical ethics. My attempt here is to analyse the relationship between the concept of happiness/well-being—certainly difficult to operationalize, it should be noted—and the states of the external world and the brain events related to (or, in some hypotheses, causally responsible for) its occurrence. In fact, outlining the different degrees of realism linked to different conception and approaches to happiness has the (increasingly relevant) purpose of providing a theoretical and practical guide to assess them in terms of consistency and of ability to offer the means to deal with the world as it is. Recent reports, which have gathered much attention from academia and the media, have tried to move the focus from the external and objective conditions “causing” happiness to conditions internal to the individual, which are therefore subjective and unrelated to states of the world (cf. Davies, 2015).

Such perspective is far from new (there are several examples of it in the history of philosophy), but today it appears to be particularly relevant due to the joint action of theoretical/psychological tools and neuroscientific ones. In a way, as we shall see, subjective happiness—being measured and induced by acting on the brain—becomes “objective” for the first time, thanks to contemporary science. In other words, it is now referred to a material and manipulable reality. The price to be paid is that happiness thus seems to be a potentially solipsistic condition à la “brains in a vat,” completely detached from the external states of the world as happiness-makers. One condition is not necessarily better than the other, but to highlight (both empirically and conceptually) the degrees of realism of these states of happiness (“natural” vs. induced) and the different means to produce them seems relevant to evaluate them along those lines and, consequently, better choose among them. And that's what I will try to do in what follows^1^.

## The different meanings of happiness

The concept of happiness has a philosophical history so long and complicated that I must necessarily introduce some simplifications and stipulative definitions, functional to the present discussion. This should not affect the reasoning to follow, since happiness will only be discussed in its meanings specified below, which are at least part of the widely recognized sense of the word (cf. Haybron, 2011). So, one can distinguish first of all an evaluative sense of happiness, understood as individual well-being, or human flourishing. Then there is a descriptive sense of happiness, at a psychological level, similar to concepts that indicate (mental/ brain) states such as “depression” or “tranquility.”^2^

### Happiness as well-being

The first meaning concerns a “prudential value” (to distinguish it from moral or esthetic values), also called “well-being” or “utility.” Such a meaning carries with it the idea of positivity and original preferability: It is better to be happy than to be unhappy—although there is wide disagreement on what it is to be happy and what means are legitimate and/or recommended to achieve this condition^3^. Happiness in this sense concerns what benefits an individual, makes her feel better, serves her interests and goals and, ultimately, is *good* and desirable for her. This characterization shows, *prima facie*, a strong subject-relative component in that what is good *for me* may not be good *for you*. If our “prudential values” are different, we can derive happiness from very different experiences. So we may think that the adventurous life of Richard Branson is what made him happy, but such life would not be as positive for, say, a monk withdrawn into the desert, happy to spend his days in meditation. In the same way, it may be assumed that a life dedicated to financial speculation is sad and disgraceful, notwithstanding the economic success it leads to, while those who do that might be extremely happy about it.

The theories of happiness as well-being can be declined in three versions: Hedonism, desire theories, and objective list theories (see Parfit, 1984). The principal thesis is considered to be that of “hedonism,” whose modern formulation, as is well-known, can be attributed to Bentham (1789). For him, nature has placed us under the sovereignty of two masters, pain and pleasure; and it is only on the basis of such masters—he argues in *An Introduction to the Principles of Morals and Legislation*—that we decide what we should do. The substantive question of the well-being implied in hedonism—in other words, when a state can be considered as a state of happiness?—is answered as follows: The situation in which the sum of pleasures is greater than the sum of pains. And the explicative question of what makes pleasure good (or a good), and pain bad (or an evil) is answered as follows: It is the pleasantness of pleasure and the painfulness (unpleasantness) of pain.

The point is that hedonism is about what is good *for me*. However, in the case of pleasure, which seems appreciated by *most* people, “for me” does not necessarily mean *only for me*. In fact, Bentham says that the central aspects of the experiences of pleasure and pain are their duration and intensity, which tend to be measurable and comparable intersubjectively. This formulation of utilitarianism is controversial, but it is not a matter that should be addressed here; suffice it to say that to mitigate its most contested aspects (for example, the fact that it would be consistent with it to prefer to live indefinitely as an oyster rather than 80 years as a fully realized human), John Stuart Mill proposed to introduce a third property, “quality,” which is to distinguish the nature and the preferability of some pleasures over others.

Desire theory regards obtaining what we aspire to, rather than the mere occurrence of certain physiological experiences. The best example can be found in welfare-based economic theories that see welfare as the satisfaction of preferences, the content of which is revealed by the individual agents in the market, who order them according to utility functions. The latter can be evaluated according to their degree of preference with the unifying meter of money. Finer distinctions are linked to actual desires (which may be dysfunctional in the long term) and to overall desires throughout existence. In any case, this perspective incorporates a high rate of subjectivism, since preferences are usually the result of personal biases, individual lives and idiosyncratic assessments, although influenced by education, culture and the intersubjective rules to which one is exposed.

Finally, objective list theories, mainly inspired by the Aristotelian position (see Hurka, 1993), regard the perfecting of human nature as the cause of *eudamonia*, a virtuous life that gives us well-being regardless of our personal dispositions, making reference to objective goods implied by human nature as such. The objection is that the list can be filled with everything that seems to produce well-being for the person in question. Thus, the supposed objective character of this approach is lost in the subject-relative dimension of the means that give us well-being. On the one hand, it's true that Aristotle seems to list a number of objective goods; on the other hand the list is actually quite loose and open-ended. “He is happy who lives in accordance with complete virtue and is sufficiently equipped with external goods, not for some chance period but throughout a complete life” (Aristotle, 2000). Indeed, those goods include health, wealth, knowledge, friends, etc. Moreover, happiness is the exercise of virtue with a balance, or a mean between excess and deficiency. Happiness thus depends on a moral character, which requires the virtues of courage, generosity, justice, and friendship. All of the above, though, seems to be referred to an individual assessment of the right set of goods and the just mean in the exercise of virtue. And this is not as objective as it should be within a realist theory of happiness, as the goods needed for one's virtuous activity depend on personal conditions.

### Happiness as a (neuro-psychological) state

The second sense of happiness is called “descriptive.” In fact, at any time any person can be characterized by a position along some dimensions that are not necessarily relevant for the person in question. This second sense of happiness can be decomposed into two levels: the classic one, which may be investigated by means of direct subjective reports or through secondary indicators of satisfaction—surveys that indicate, for example, that Londoners are on average happier than Liverpudlians—and the neuroscientific one, which has to do with the observable and measurable activation of the brain areas found to be associated with pleasure and states of well-being. This objective and quantifiable method has sparked the idea of building a “science of happiness,” both on the descriptive level and on that of the increase in individual well-being by acting with various tools on the relevant neuronal circuits (see Berridge and Kringelbach, 2011). I will call the first of these levels “subjective psychological happiness” and the second “objective brain happiness.”

Subjective psychological happiness has long been the subject of debate as for what lies beyond its intuitive commonsensical meaning, i.e., that for which we have no difficulty in saying whether we are happier today than yesterday or in ordering on a scale the degree of happiness associated to specific events or situations (ignoring for now the fact that there are strong distortions of judgment related, for example, to the nearness or the heterogeneity of the facts considered). This shows that happiness is a concept easy to understand, that, however, has raised the difficult question (even existential, as shown by millennial wisdom and religious reflection) as to what are the mental states that correspond to happiness (if there are any).

The main candidates for “descriptive” happiness, according Haybron (2011), are non-welfare hedonism (physical or immaterial pleasure, the latter being also, we now know, largely mediated by the same brain mechanisms involved in bodily sensations), life satisfaction theories and emotional state theories (where the state is to be understood as positive). The last two have to do with a disposition, an orientation toward the conditions of one's own life: what matters is not so much the experiences that happen to us but the way in which we accept and evaluate them, by emphasizing our emotional side in one case (thereby also involving emotions at the cerebral and physiological levels), while leaving room for a more thoughtful and rational judgment in the other.

The hedonistic position implies that happiness corresponds to the balance between the sensations of pleasure and pain, although it is not considered that that balance should be the only concern for each individual. On the other hand, the theories of “life satisfaction” describe happiness as a favorable attitude toward one's life taken as a whole, a sort of global assessment that is not only a theoretical or intellectual consideration, but means embracing and asserting one's life as such, in a way that may be partly implicit and may or may not be accompanied by some emotional features.

Finally, the emotional state theory does not consider the feelings of pleasure but rather the constant emotional background that accompanies us, which is a persistent tone and is not subject to continuous deviations depending on individual daily events. This is primarily a state of mind that we would call “serenity” and “contentment,” unrelated to specific events or situations. It's something that, ultimately, one might consider to be the opposite of anxiety and depression, emotional states that are more easily identifiable (Haybron, 2008).

## Happiness and realism

Even if happiness, as stated, may seem like an elusive state of the individual, something private and therefore evasive of any objective analysis, in this paper I am examining attempts to find operationalizable definitions and quantifications of it. In this context, empirical studies began with the construction of indicators and scales for measuring personal and inter-personal happiness. It was also discovered that subjectively reported evaluations are very often correlated with relevant objective variables, such as friendship, physiological data, health and longevity (still leaving open whether they are among the causes of happiness—which is yet to be defined—or whether they are part of the state called “happiness”).

This is where the element of realism comes into play, since objective variables are mind-independent and intersubjectively observable. If we abstract from the radically subjective sense—so that everyone can say when and how one is happy in a sort of Wittgensteinian private language in which nothing can be said that is scientifically and philosophically interesting—it is possible to introduce the criteria of science and naturalism so as to test if and how their inherent realism is relevant in such a particular context. There is indeed a long tradition that has theorized the overwhelming superiority of the inner perspective, i.e., the idiosyncratic and personal look at one's individual happiness. But the concept of happiness remains very complex with the combination of internal and external elements that it implies.

### Descriptive states and prescriptive theories

It should be pointed out that in the discussion of happiness, and particularly in its relationship with the realism/anti-realism debate, we can distinguish between a descriptive level and a prescriptive level. The first one says what happiness is and what its cause is, while the second concerns the possible ways to achieve happiness, going beyond the state in which one finds oneself and identifying the key elements upon which to act. As that of happiness, so far, has not been an exact science, both areas are characterized by empirical approximations and generalizations drawn from experiential accounts, regardless of the fact that one can be “mistaken” about one's state of happiness, unlike what happens with the attribution of one's subjective phenomenal states.

#### Hedonism explained by neuroscience

However one philosophically defines hedonism and its place in the economy of happiness, recent progress in the scientific understanding of the physiological mechanisms of pleasure seems to break down the boundaries between types of pleasures usually held to be totally incommensurable (e.g., food and intellectual achievements). This widens the perimeter of hedonic states as a subset of happiness, through an at least partial integration-overlapping with eudaimonic states. Hedonic states are those that fall within the theory of happiness as well-being and, in the descriptive states, as objective cerebral happiness. Eudaimonic states are those referred to both by the desire theory and by the objective list theory, namely the cognitive and moral aspects that are part of a life considered good and significant (the two dimensions, however, tend to correlate in subjective measurements).

Pleasure is never made up of a simple feeling: it always needs the active involvement of specialized brain systems responsible for the hedonic coloring of individual sensations (Berridge and Kringelbach, 2011)^4^. The activation of small portions of our brain is what makes us pleased by the individual experiences that have the ability to cause such cascades of electro-chemical processes. These mechanisms, with different degrees of complexity, are found throughout the animal kingdom, indicating that their origin is filogenically ancient and evolutionarily adaptive. Beyond food and sexuality, social interactions are also sources of pleasure, as well as—it seems—the positive feelings coming from personal or creative achievements^5^.

Although the feeling of pleasure can be unified, subpersonal mechanisms in action constitute a complex structure composed of several components of the brain, which with their selective recruitment and their activation of different intensity give rise to those that—at the phenomenal level—appear as hedonic states of different origins and of different types. In fact, what cognitive neuroscience seems to tell us with increasing accuracy is that everything is mediated by the same processes and that pleasure is a creation of the brain largely independent from the origin of its stimulation^6^.

This fact implies that the apparent extreme subjectivity of hedonic states—long believed to be due to the different (and unfathomable) physiological response to an almost infinite variety (both for quality and for quantity) of potentially enjoyable elements—is no longer in contradiction with the objectivity of the physiological response (in its translation at a conscious level) of single individuals. This could be inferred from the evolutionary explanation: if the perceived pleasure must serve to guide adaptive behavior, foods rich in lipids, and glucose (the rarest in the diet of our hunter-gatherer ancestors) must be those that give to all the specimens the greatest pleasure, so that it acts as an incentive and reward for the effort to get the most nutritious and useful food in order to survive and thrive in the environment.

But the latest research has gone further. The first step was the identification of the correlation between the activation of different areas—evaluated with the tools of neuroimaging—and behavioral indicators of satisfaction caused by specific stimuli. An example, for animals and human infants, are the facial expressions of happiness and the protrusion of the tongue aroused by substances with a sweet taste. On the contrary, bitterness induces expressions of disgust, grimaces, head movements, and the expulsion from the mouth of the substance ingested. In such cases, there is no cognitive mediation, or learning (the child has not yet learned that it is not appropriate to spit), but only the immediate and automatic reactions dictated by the feeling that causes the activation of certain brain areas related to chemical detection of the receptors placed on the tongue.

A next step that has been taken is the hyperstimulation of the brain areas thus identified so as to verify whether there is a causal relationship and not solely a correlation between their activation and a hedonic feeling. In rodents, microinjection of molecules that mimic the neurotransmitters implicated in the transmission of impulses in the areas subjected to the experiment have actually led to a strengthening of the observable reactions of pleasure or satisfaction after the administration of substances with a sugary taste (Berridge and Kringelbach, 2013).

This seems to indicate that the brain mechanisms are the final and most important elements responsible for conscious hedonic feelings, given that (as it was previously thought) in the same situation and in the same specimens a sugar solution could have caused greater or lesser satisfaction according to the endogenous microstimulations. And, again, this shows how it is possible in principle and, today at least in part, empirically viable to arouse in the brain the same pleasure that would give us, say, a chocolate bar or a hot bath, with a direct stimulation of the brain.

The precise identification of the areas involved in the generation of conscious sensations of pleasure and the ability to stimulate them with increasing accuracy, as well as the measurement or at least the operationalization of subjectively experienced pleasure, can be seen as an advancement toward the arithmetic of pleasure suggested by Bentham. Pleasure, then, seems to change from subjective and elusive to a completely understandable process at the scientific level, with an objectivity that, therefore, cannot be contested. Remember, however, the possible objection linked to the still incomplete understanding of the way in which the molecular processes translate into conscious phenomenal sensations for the individual (the so-called *qualia*).

Futuristic scenarios (see Linden, 2011) indicate the possibility of finely manipulating the brain circuits of pleasure: a person who took advantage of such a device could experience the same hedonic states as those elicited by stimuli coming from the outside of her brain. On the other hand, philosophers had already made the same mental experiment. Nozick (1974) has famously conceived a device where people could come and experience all the mental states corresponding to experiences in the outside world.

Leaving aside the arguments that show how we cannot be “brains in a vat” without realizing it (Putnam, 1981), Nozick had knowledge at the time of writing that led him to claim that there is something else that “we want more than our experiences” and, in particular, “we want to do certain things, and not only have the experience of them.” But according to contemporary neuroscience there is no difference, at least potentially, between remembering to have taken some action in the world, with the associated feelings, and remembering a stimulation produced directly into our brains.

In any case, here I am only referring to states of pleasure and not to the whole life experience. The hedonic component is therefore measurable and reproducible material, realistic in terms of its scientific description, which identifies the causes and effects according to the nomological regularities of biology (even though biology is not an exact science). Happiness understood as well-being resulting from pleasant experiences is therefore fully part of the field of realist ontology independently from subjective evaluation. It is a construction of our brain as a material organ, which responds to stimulation of certain parts of it (however they are produced) assuming different physical states over time (t2) and which following stimulation with respect to time (t1) is characterized by a different stimulation (or by no stimulation at all).

One can therefore agree that hedonic states can be classified as “realistic,” rooted as they are in an object (the brain) that is fully part of the “furniture” of the world, in contrast to the strongly subjectivist perspective which has always characterized them as ineffable and mind-dependent. The realism of the hedonic states thus described may be termed “internal” (in a peculiar sense, which does not refer to other uses of the term in the philosophical debate), as it regards modifications unique to the individual brains. The status of other theories of happiness seems to be different.

#### The desire theory

If that of hedonism is an internal realism, desire theory—which concerns the obtaining of what we aspire to, rather than the mere occurrence of certain physiological experiences—has a character of “external” realism (again in a particular sense, unrelated to earlier uses of the expression). In fact, the states of satisfaction are anchored to states of affairs in the world separate or independent from the individual, although their evaluation as good/happy is partly subjective, related to “culturalist” elements, i.e., rooted into a specific cultural niche made up of consolidated habits and traditions. Think of the distinction established by Dworkin (1993) between *critical interests* (that is, general purposes even external to the self: the well-being of children, the results of one's work in relation to the community and so on), as opposed to *experiential interests* (related to momentary personal satisfaction although not to the simple pleasure that we have called “instinctive”). It appears that the former do not pass Nozick's experience machine test.

If the well-being of our children is what we want in a particular way, we will definitely have a *sui generis* hedonic state associated with the realization of this kind of well-being, which can be simulated by the device that connects to our brain to stimulate certain areas; but what we want is that our children *are* in good health, that they *are able* to start a family and to achieve their professional goals, that they *do not suffer*. And we want these things to happen *in the world* and not in a simulation that will give us the pleasant (for us) illusion that they're doing fine, while maybe they are dying in a hospital. In this case, it seems that there is a clear separation between the implementation of possible (though real) states of hedonic brain satisfaction and the appreciation of states in the outside world as the conditions for happiness tied to the external realism of critical interests^7^.

On the other hand, experiential interests are fully included in the hedonistic theory of happiness, so that pleasure can be aroused by external stimuli as well as by endogenous activations, but the link with the states of satisfaction experienced subjectively—with the exception of the cognitive-moral component that we will consider later—does not necessarily depend on the external states of the world, but rather on the particular internal neuro-physical state (the absence of a certain type of sensory receptors can deprive us of the intense pleasure that a given food arouses in most of our fellow human beings).

#### The objective list theory

The objective list theory, as I said, refers to the improvement of human nature as the cause of *eudemonia*: a virtuous life can give us well-being regardless of our personal dispositions, as it refers to goods independent from subjective evaluations, implicated by human nature itself. It is relevant here to recall the objection that Nozick himself makes against the experience machine.

A second reason for not plugging in is that we want to *be* a certain way, to be a certain sort of person. Someone floating in a tank is an indeterminate blob. There is no answer to the question of what a person is like who has long been in the tank. Is he courageous, kind, intelligent, witty, loving? It's not merely that it's difficult to tell; there's no way he is. Plugging into a machine is a kind of suicide. It will seem to some, trapped by a picture, that nothing about what we are like can matter except as it gets reflected in our experiences. But should it be surprising that what *we are* is important to us? (Nozick, 1974; p. 43).

The happiness that comes from being a certain type of person has to do with the intersubjective—objective, external, verifiable—recognition of some parts of our personality that must result (at least in part) into recognizable actions or affections. There is certainly the extreme case of the solitary ascetic. But even if the virtuous life does not seek publicity, it is still inserted into the reality of the world because, according to Aristotle, it recognizes an external order to follow (Aristotle, 2000). The objective list theory can also be descriptive because, in the view of its advocates, there is an inherent tendency in people to prefer certain goods and to have cooperative attitudes toward their peers, in a virtuous circle of individual integrity and social benevolence. What was a philosophical reflection has now become evidence of empirical and evolutionary psychology and cognitive neuroscience, thanks to the discovery of mirror neurons and brain circuits of empathy.

### Subjective psychological happiness

The description of happiness includes the life satisfaction theory and the emotional state theory. They seem to be mind-dependent, in principle detached from the states of things external to the subject and, therefore, they can be defined as interpretative judgments lying beyond objective facts (a position à*la* Nietzsche). Obviously, psychological states have a cerebral correlate and changes in brain states seem to affect (if not the existential evaluation) at least the emotional state. It is known since the dawn of mankind that psychoactive substances have the ability to change one's mood, also altering one's evaluation of one's condition and of the states of the world.

It should be noted that “assessment” is not understood here as a purely cognitive judgment that may be distorted under the influence of drugs—such as the estimation of the width of a room—but the emotional-cognitive appreciation of complex situations, such as the desirability of a certain conduct or of a certain existential situation: for example, whether to quit a job in which one undergoes mobbing or not. The inability to make important decisions on the part of individuals who have suffered injuries to specific brain areas that govern the elaboration of emotions—as shown in studies by Damasio (1994)—comes from the lack of indicators of the salience of the alternatives provided by the physiological emotional response.

On the other hand, if it is true that we now have highly selective molecules to modulate the mood and, consequently, the emotional state of a person, we still do not know exactly how they work. Prozac, the psychiatric drug that defined a social era (see Kramer, 2006), making depressed patients feel “better than good” and paving the way to the age of “cosmetic pharmacology,” increases the levels of the serotonin neurotransmitter available between certain target synapses, but it does not seem that this effect is sufficient to induce those changes of personality that bring people under treatment to see their life in pink. Serotonin reuptake inhibitors, however, are candidates to also affect life satisfaction.

But what is relevant in the descriptive state of happiness-psychological euphoria—beyond the substrate of “internal” realism yet to be deciphered (and therefore not reproducible by the experience machine, because it is different from the states produced by hedonic pleasure centers)—is its deep irrealism. This aspect is captured effectively in a recent novel that combines literary sensitivity and scientific expertise. The protagonist of *Generosity* by Powers (2009) fascinates those around her because of her contagious happiness, which consists in a really good mood (which can be measured by psychological assessment tools) and in a totally positive look on reality. The joy of living in Thassa Amzwar, a self-conscious and intelligent girl, appears disconnected from her life history (her beloved parents were killed, she had to leave her country because of the civil war, she is an Arab immigrant in Chicago; albeit educated and multilingual, she has uncertain prospects for her future, a classmate she got close to tried to rape her…). Her amazing and contagious optimism arouses the interest of some researchers, who discover that the secret of Thassa's happiness is enclosed in a rare mutation in her DNA. The discovery triggers collective hysteria in the hope of creating a technique of genetic manipulation capable of giving perpetual euphoria, while the young woman sees her non-superficial joy waver for the first time.

The ability not to break down and not to fall into inaction as a reaction to the events that affect us is certainly a valuable adaptive resource. It is a result of many studies that people have a mid-point of happiness they tend to come back to after peak events, both positive and negative, such as winning the lottery or surviving a serious car accident. At the present state of knowledge, it is estimated that 50% of subjective well-being is hereditary: such data emerged from studies on twins (cf. Brickman et al., 1978; Fredrickson et al., 2013; Bhattacharjee and Mogilner, 2014).

Nevertheless, those of “life satisfaction” and emotional state are theories and conditions of happiness that can have a very high rate of irrealism, understood as a mind-dependent cognitive state, which does not correlate with the objective facts of the external world. This happens when one can be happy or sad facing the same situation (for example, having lost a rewarding job and not being able to find another one); and when the correlation between the state of the world and one's own subjective state looks quite idiosyncratic, seeming wrong or dysfunctional to most observers, (for example, if the servant declares himself happy to be chained). Thus, the lack of realism about happiness can have serious consequences on the objective quality of life, which are different from the simple fact that it seems better to be moderately happy rather than mortally depressed (Oishi et al., 2007).

### Prescriptive theories

The topic of realism associated with the discourse on happiness becomes burdened with practical consequences when we enter the domain of prescriptive theories, that is, theories that advocate a sense of happiness as better or preferable and indicate the manner in which to achieve the highest and most enduring states of happiness. The states of satisfaction that each individual decides to pursue are, of course, the result of subjective judgment (which could in turn be the effect of an objective cerebral asset), with a non-idiosyncratic and non-relativistic component. A meta-ethical discourse on happiness, however, is not my focus here. I will therefore consider approaches that do not blatantly violate the basic rules of the law and common ethical sensitivity.

Hedonism is the approach that sees happiness as equivalent to pleasure. Empirically, today we might limit it (or perhaps, better, extend it) to all the states generated by the pleasure areas identified in the brain, whatever the stimulus (endogenous or external) that causes them. Nozick's experience machine is fully part of the hedonistic recipes for happiness, just like drugs, as long as they give us pleasure.

#### Is realist hedonism also naturalist?

Hedonism is a form of “internal realism,” because pleasure can be identified and quantified with the experimental method much more than a subjective feeling, while also being a subjective feeling. Internal realism, however, is not necessarily linked to the outside world; it can, indeed, lead to detachment from the world and one's own critical interests, if they are rooted in states of external reality. The laboratory rat that keeps stimulating itself by moving a lever that controls an electrode implanted in its brain and those who compulsively seek inner satisfaction in food, sex, or even in solipsistic intellectual gratifications are united by a distancing from the states of the world beyond their own hedonic inner reality—as evidenced by the fact that all of them would probably agree to be connected to Nozick's experience machine.

Provided that naturalism (the notion that all existing things are physical and are as described by science) is not prescriptive and, on the contrary, it has a hard time finding its place in the physicalist ontology for its normative dimension (“What should I do?” “Who is a better poet, Chaucer or Donne?”), hedonism as a quantifiable description of brain activations that give pleasure can be defined as naturalistic realism. In other words, scientific naturalism is only committed to describing nature and reality as they are, not to finding the way nature and reality should be—since there are not principles or values detectable by science in accord to which establish what happiness should be—or what behavior one should adopt (i.e., the so-called normative dimension). (Obviously, scientific naturalism is not the only naturalism on the market, and other forms of “liberal” naturalism can accommodate moral values and rules; but those forms of naturalism are often disagreed with).

#### Irrealism in positive psychology

“Have these two thoughts ever the readiest in all emergencies: one, that ‘the things themselves reach not to the soul, but stand without, still and motionless. All your perturbation comes from inward opinions about them.’ The other, that ‘all these things presently change, and shall be no more.’ Frequently recollect what changes thou hast observed. The world is a continual change; life is opinion.” Thus says Marcus Aurelius in *Meditations* (4:3), and seems to echo the Buddha when he says: “All mental phenomena have mind as their forerunner; they have mind as their chief; they are mind-made. If one speaks or acts with a pure mind, happiness follows him like a shadow that never leaves him” (*Dhammapada*, verse 2). According to Haidt (2006), the most important idea of folk psychology is contained in these two quotes: world events affect us only through the interpretation that we give of them, so if we can control the interpretation, we can also control our world. So much so that a guru of folk psychology has coined one of his 10 laws so that it echoes Nietzsche's position: “There is no reality, there is only perception (Haidt, 2006).

Handbooks of self-esteem and self-help have the specific aim of changing these interpretations so as to make sure that the individual becomes happy not by acting on her situation in the world, but by adapting to the world with a change of perspective that makes her understand the positivity of their condition, once it has been better considered. Thus, in an example given by Haidt, there often comes a time when a person worn out by years of resentment, pain and anger realizes that (say) her father did not hurt her directly when he abandoned their family, but merely left the house. It is the reaction to the event which gives rise to pain—according to this reading—so that if we abandon this reaction, the fact itself cease to be a source of suffering.

Giving different judgments on facts is not the same thing as denying reality, but it is often a strategy that can get close to denial if moral judgment is strongly deformed, or when there are no specific reasons to try to change our ordinary intuitions about reality instead of changing the states of the world that seem negative or dysfunctional. It is a different thing compared to what is called “average affective style,” a way of emotionally assessing events that happen to us that, based on what Haidt effectively calls “cortical lottery,” can be a constant bad mood making existence a heavy burden. In the long term, meditation, psychotherapy, or medications such as Prozac can be effective on the affective style. But here we return to internal brain realism, because it is possible to think that all these interventions, mediated or direct, act on some neural circuit deputed to the development of emotional evaluations^8^.

The position of at least part of positive psychology is quite different, as it is strongly tempted by the irrealist perspective. Ehrenreich (2009, cf. also Dawes, 1994) pointed out that positive thinking distorts reality, so as to only see its favorable factors, or simply denies it. This trend can encourage people to submit to adversity with a benevolent disposition (“cancer makes you better, it is an opportunity to change”) and blame themselves for the blows of life (“if you do not recover from cancer it is ultimately because of your negativity,” as if cancer were a psychological condition). If we have cancer we must seek proper care, which sometimes is not available, and a stoic or optimistic attitude can be surely useful and admirable. But in front of other non-unavoidable events, such as social injustice and political oppression, we may end up passively accepting unjust situations as a result of the pursuit of inner happiness. Seeking “life satisfaction” in spite of the external states around us seems to amount to an illusion that distorts the evaluation of oneself and reality.

Even the so-called humanistic psychology of Abraham Maslow and Carl Rogers, influenced by the subjectivist-spontaneistic cultural climate of the 1960s, has contributed to an optimistic relativism that promotes inner realization and the growth of one's inner potential, with the idea of harmonizing the self with the cosmos and not vice versa (see Milton, 2002). Some simplifications of anti-psychiatry have led to believe that there are no objective diagnoses and that inner needs precede, and may be manifested regardless of, the external situation.

As I said, it is not in question whether happiness should be the goal of existence, or whether we should rather prefer a “good” or “truthful” life, adhering to mind-independent realism. Even in the pursuit of one's own well-being, to consider the relevance of both internal states and the external states of the world is important. One could ask whether the happiness of the wise à*la* Epictetus, who knows how to cut himself off from the worries and cares of the world, is a form of unconscious irrealism driven by false beliefs, induced or self-inflicted, or a form of selfish solipsism, which favors one's own inner satisfaction at the expense of a possible engagement in the world.

The recent trend that combines positive psychology with research on the neuroscientific bases of specific mental states can be placed in this area. It started from the benefits of contemplative practices and got to the “contemplative neuroscience” also promoted by the Dalai Lama, who first lent himself as a subject of direct study (Ricard et al., 2014). The discovery of the benefits of meditation is in fact associated with neuroscientific advances that have shown that the brain can be transformed, and brain circuits can be rewired, by the experiences to which it is exposed. In this case it comes to internal experiences, by definition disconnected from the external ones, which are considered sources of disturbance and anxiety. It's mainly about “focused attention” and “mindfulness.” The first leads the meditator to concentrate on the in-and-out cycle of breathing. The second entails observing sights, sounds and other sensations, including internal bodily sensation and thoughts, without being carried away. Mindfulness makes a person “aware of what is happening without becoming overly preoccupied with any single perception or thought, returning to this detached focus each time the mind strays” (Ricard et al., 2014; p. 42). Neuroscientists have tried to measure mindfulness meditation and found that it produces decreased sensitivity to pain, as well as a reduction in symptoms of anxiety and depression. The neurocerebral study of people who have a long experience of meditation, especially people of the Buddhist religion, has shown that they are able to sustain a Particular EEG patterns, in particular the so-called high-amplitude gamma-band oscillation and phase synchrony at between 25 and 42 Hz. The subjects have different EEG traces (Lutz et al., 2009). Also, it seems that mindfulness training can decrease the volume of the amygdala, a brain area considered crucial for the development of feelings of fear and anxiety. In this way, it is shown that the “meditation produces significant changes in both the function and structure of the brains of experienced practitioners” (Ricard et al., 2014; p. 45). This is important because, for the supporters of mindfulness and positive psychology in general, “people differ in their levels of happiness, and these differences are associated with different underlying characteristics” (Davidson, 2005), “emotion regulation plays a key role in modulating these differences in happiness,” and “happiness can be regarded as the product of skills that can be enhanced through mental training, and such training can induce positive changes in the brain”(*ib*.) (cf. Lewis et al., 2014).

The consequence is that happiness can be increased by acting on the above-mentioned skills through a proper and systematic training (Ricard, 2006). This goes in the direction of positive psychology (Seligman, 2011), for which personal flourishing (which replaces happiness) by definition has no external criteria to decide what accomplishment is valuable to someone. This leads to an individualist trend that only marginally considers the happiness of the group. The risk is to undermine, in the long run, the very bases of social coexistence and rewards, which are the preconditions for any real flourishing. In fact, if we detach the pursuit of subjective happiness from external intersubjective criteria, the result could be forms of quietism weakening social bonds and collective cooperation, which are necessary for the maintenance of the material and social standard we have today. Alternatively, such pursuit of happiness might lead to a gap between individual values and purposes able to interfere with the possibility of having stable bonds and relation at a community level.

The latest stage of this trend has been reached by recent devices promising to make their users' happier (Davies, 2015). For instance, one of them, thanks to sensor-laden headband which monitors the neuronal activity, should help improve the emotional state of the user, thereby training the brain for empathy and composure. But to “mechanicize” happiness through cerebral manipulation, albeit freely chosen, will inevitably lead to irrealism with regards to the external states of the world and to (more or less) objective criteria of happiness understood as more than an EEG pattern. In fact, these devices might be used to avoid the ups and downs of real life, which help us find our way in the social and physical world. The ultimate effect may be an estrangement from one's context and acquiescence to the given situation, with no incentive to change dysfunctional situations for one-self and for others or to improve one's own or the others' conditions. This is a situation of extreme irrealism, according to my definition, even though happiness as it is subjectively experienced and neurophysiologically detected is here perfectly “real.”

#### The realism of the theory of capabilities

A theory of well-being that aims to avoid the trap of irrealism is that proposed by Amartya Sen and Martha Nussbaum, called “theory of capabilities.”^9^ The overall objective of this approach is a society in which everyone is treated as worthy of respect and put in a position to really be able to live in a humane way. The key point is that below a certain level of capability a person is not able to live a truly human life (Nussbaum, 2011). It is a theory that does not want to be culturalist. Therefore, while some individuals may be say to be happy in their condition (e.g., baby-prostitutes in the slums of Calcutta, as some studies indicate), they will not live a truly human life: in those cases, they deceive themselves about their happiness, through a representation of it that is not realistic, maybe because they do not have sufficient knowledge or the right tools.

The list of capabilities is not based on subjective impressions of satisfaction, but on objective conditions: it is those human capabilities that give dignity and well-being, and can serve the general purpose of every human life, regardless of the specific goals people aspire to achieve. Basic capabilities, however, are not just tools; they are intrinsic values to give a properly human character to existence. For Nussbaum they include: life, health, bodily integrity, senses, imagination, and thought, emotions, practical reason, affiliation, other species, play, control over one's environment. Their identification is carried out by means of a widespread and negotiated consensus, which refers both to the universalitic idea of humanity and to mind-independent elements that play a real role in people's lives.

It can be argued that the welfare of truly human existence in the perspective of basic capabilities is not happiness as we usually understand it, which is the state that stands out *above* the usual average of our states. As already mentioned, a person can be said to be satisfied with their life or appear to be so to many observers even if they happen to be in a condition that seems (according to the observers or to shared criteria) unpleasant, painful or not emotionally satisfying. These people are well below the minimum threshold of capabilities that the theory sets for humanity, not even for happiness. The idea therefore is that in the absence of alternatives or opportunities we tend to a psychological settlement that spares us excessive suffering, but that we could come to a “real” and better condition, according to criteria that once experienced we would share: this would give us greater satisfaction and “more real” happiness rather than the mere result of “interpretations.”

In any case, it is interesting to note that, with an independent path, Williams (1985, ch. 9) has also come to recognize a kind of realism in the idea that there might be a theory of human nature, based on the knowledge provided by the social and physical sciences, capable of guiding a reflection on what leads to flourishing. That is, while maintaining the relativism of values, there can be objectivity in the scientific sense (objectivity that creates a convergence as a mirror of reality) about the belief that a certain kind of social world is the best one in which human beings can live.

## Conclusion: Degrees of realism

Contemporary neuroscience seeks to explicitly describe with its quantitative methods (including predictability) an elusive—although somehow operationalized—concept like happiness, not only in its hedonic component, but also in the eudaimonic one (which is believed to also stem from brain processes). It is a powerful operation by which what has always been considered a stable condition, predominantly psychological, pervading consciousness, and deriving from the total satisfaction of the inclinations and desires of the human being, is reduced to a strictly naturalistic condition. This is a good chance for a renewed confrontation between realism and subjectivism-culturalism—that is, exactly the comparison that new realism proposes to establish.

As I have tried to argue, there are different degrees of realism related to different conceptions of happiness. The first is what I have called “internal realism,” which is also naturalistic, being fully objective and mind-independent (leaving aside here the non-secondary element of *qualia*). It is closely related to the functioning of our brains so, in this sense, it is independent of the states in the outside world, which are often responsible for feelings of pleasure. It may be pointed out here that the evolution of the hedonic brain structures has certainly been driven by a feedback coming from the environment, since pleasure is originally a signal of the “appropriateness” of the specific relationships between the body and, for example, some type of food or the chosen sexual partner. As emerges with Nozick's experience machine, though, such a connection is not necessary and, indeed, the most promising scientific perspective is that of simulating and increasing pleasure by only acting on the brain.

The second level of realism is called “external,” and has as a prerequisite the states of the world, which are *per se* the cause of happiness or unhappiness. This would still be the case even if one were to discover in the near future the internal “mental” mechanism that makes it possible for such states of happiness to be in any way comparable to that of pleasure in the classic sense. A third type of realism related to happiness is a metaphysical one, of a natural objective order, mind-independent, and reachable with non-naturalistic faculties. This type of realism is typical of rationalist theories.

There is also a form of irrealism about happiness, namely the one represented by a very wide and ancient tradition that identifies eudaimonic states with detachment from the world and the focus on oneself (but not in the sense of rationalist theories), in an attempt to passively escape the pain that the world can cause. The maximum degree of irrealism is found in all those prescriptive theories suggesting that one should change one's opinion on reality in order to represent the world in subjectively positive terms, regardless of the actual states of the world. The same holds for the kind of happiness that, according to empirical psychology, many people claim to experience even while being forced to live in conditions of hardship, misery, and exploitation. According to a strictly naturalistic approach, this may be due to the “cortical lottery”: depending on their genetic asset, some individuals have a higher average level of happiness then their fellow people, to which they return after each peak, be it positive or negative.

This reading effectively indicates the difference between internal and external realism: if for some it is internal brain mechanisms that determine happiness no matter what happens “out there,” there is an obvious disconnection between the reality of the biochemical structure of the brain and the reality of the states of the world external to the subject in question. (Of course, there are general cognitive resources to understand this disconnection). A test of realism with respect to these situations is given by theories such as that of capabilities or that of “primary goods” by Rawls (1971), which don't consider subjective judgments, but rather some kind of minimal shared human flourishing. Such flourishing is very different from that of positive psychology, because the latter is largely dependent on the subjective emotions of the individual, while that described by the capabilities theory is anchored to objective criteria resulting from an intersubjective rational weighting, which is subject to confirmations and corrections in time.

In the end, scientific realism seems to contradict the well-known saying that “everyone knows what's good for them,” understood as the impossibility for others to assess the degree of happiness that other people experience. Nonetheless, individuals should be able to choose for themselves what type of happiness is the most appropriate. However, this choice is not morally indifferent, as it affects what kind of life we want to live (and perhaps also what kind of person we want to be) as well as the fate of others, as I have tried to show so far. Knowing the different degrees of realism of the idea of happiness one adheres to or publicly promotes is therefore strongly relevant and is surely one of the tasks of the psychological and philosophical reflection.

## Author contributions

The author confirms being the sole contributor of this work and approved it for publication.

### Conflict of interest statement

The author declares that the research was conducted in the absence of any commercial or financial relationships that could be construed as a potential conflict of interest.

## Footnotes

^1^It should be noted that by “realism” I mean the adherence to an objective state of things in the world. In general, realism can be so defined: X, Y, and Z exist, and the fact that they exist and have specific properties is (ontologically) independent of our beliefs, perceptions, linguistic practices, and conceptual schemes. Realism thus implies the existence of a something independently of any subject's states of mind. In this sense, “objectivity” can also be used, as it generally expresses the notion that the quality of being true is not influenced by particular perspectives, individual or community biases, interpretations, and value commitments. In this sense, objectivity is an ideal for science as well. The problem here is the extent to which one can speak of objectivity when describing mental or emotional states involved in describing happiness and wellbeing. In any case, I prefer to frame my discussion along the realism/anti-realism disjunction, as the contemporary philosophical and psychological debate about realism and anti-realism seems to be more nuanced and to offer some helpful tools. Anti-realism here amounts to detachment from a state of things in the world independently of specific mental states and, in some cases, the belief that there is no objective reality to refer to. Anti-realism concerning happiness is peculiar, in that it doesn't deny the existence of a specific class of objective states of the world (the individual cerebral states underlying the mental states of happiness), but believes (or is committed to the idea) that such states are unrelated to all other objective states of the world, which can be instead their causal origins or a comparative reference when assessing well-being.

^2^I will not tackle here the issue whether one needs to distinguish between mental states and brain neurobiological structures (and if so, what relationship there is between them, if mere co-presence, correlation, or causality), or whether they fully coincide (as psychoneural reductionists claim).

^3^Here and below, I follow Haybron (2011) and Crisp (2008). There are also moral positions for which we should not give much importance to happiness. I will argue that this may be related to the degree of realism and anti-realism incorporated in the different senses of happiness assumed.

^4^Although it exceeds the possibilities and scope of this essay, the concept of pleasure should also be restricted in a more precise way. To a first approximation, it can be defined as the quality of our internal states (living beings have a continuous flow of internal states) characterized by preferability, that is, by the wish for their achievement or continuation.

^5^The central axis of the so-called pleasure circuit consists of the dopaminergic neurons of the ventral tegmental area and its projections to the nucleus accumbens, but the prefrontal cortex, the dorsal striatum, the amygdala and the hippocampus are also involved (for the details, not yet fully understood, see e.g., Linden, 2011; Bloom, 2010).

^6^One might want to consider a philosophically central theme, that of the externalism of mental contents. For hedonic states, the issue seems to be less pressing, but when it comes to beliefs the problem is unavoidable. If one adopts the position that the contents of beliefs are related to a subject's relation to her environment, internalist perspectives are radically challenged (including Nozick's experience machine when it promises to faithfully reproduce real-life experiences). However, if the proximate causes of behavior are locally supervenient in the brain (as neuroscience seems to posit) the externalist objection is called into question. It is not possible to develop this debate here, as it makes the discussion of the relationship between realism and happiness even more complex.

^7^One could object that external conditions might seem here to be regarded as constitutive factors of happiness. In fact, external conditions are constraints to a realist notion of happiness, as opposed to a subjective one. One cannot be “happy” if objective external conditions are not consistent with the realist notion of happiness. Consequently, external conditions are causes and reasons of happiness, since realist happiness is not the same thing as its conditions.

^8^It should be noted, however, that the “artificial euphoria” produced by the new generations of psychoactive drugs has been described as a “dishonest happiness” because of the escape from reality that it implies (see President's Council on Bioethics, 2003, cap. 5).

^9^Those of Nussbaum and Sen are properly theories of human development, but to the extent that they aim to promote overall well-being of the person they may fall within the scope of our theories of happiness.
